# Percutaneous thermal segmentectomy for liver malignancies over 3 cm: mid-term oncological performance and predictors of sustained complete response from a multicentric Italian retrospective study

**DOI:** 10.1007/s11547-024-01877-w

**Published:** 2024-09-16

**Authors:** Pierleone Lucatelli, Bianca Rocco, Renato Argirò, Vittorio Semeraro, Quirino Lai, Elena Bozzi, Sara Crociati, Michele Barone, Alessandro Posa, Carlo Catalano, Laura Crocetti, Roberto Iezzi

**Affiliations:** 1 Interventional Radiology Unit, Department of Diagnostic Medicine and Radiology, UOC Radiology, Sapienza University of Rome, Rome, Italy; 2 Diagnostic Imaging and Interventional Radiology, University Hospital of Rome Tor Vergata, Rome, Italy; 3 SSD Radiologia Interventistica POC SS Annunziata - ASL Taranto, Taranto, Italy; 4 General Surgery and Organ Transplantation Unit, Department of Surgery, Sapienza University of Rome, Rome, Italy; 5 Department of Radiology and Interventional Radiology, University of Pisa, Pisa, Italy; 6 Fondazione Policlinico Universitario “A. Gemelli” – IRCCS, Dipartimento di Diagnostica per Immagini, Radioterapia Oncologica ed Ematologia - Area Di Diagnostica per Immagini, UOC Radiologia d’Urgenza ed Interventistica, L.go A Gemelli 8, 00168 Rome, Italy; 7 Università Cattolica del Sacro Cuore, L.go F Vito 1, 00168 Rome, Italy

**Keywords:** Hepatic tumor, Balloon-occluded MWA, Balloon occluded TACE, Combined treatment, Interventional radiology

## Abstract

**Introduction:**

Percutaneous thermal segmentectomy is a single-step combination of microwave ablation, performed during arterial occlusion obtained with a balloon micro catheter, followed in the same session by balloon-occluded TACE. The aim of this multicenter retrospective study is to report the mid-term oncological performance of this technique for liver malignancies > 3.0 cm and to identify risk factors for the loss of sustained complete response.

**Methods:**

Oncological results were evaluated with CT or MRI according to m-RECIST (HCC) and RECISTv1.1 (metastasis/intra-hepatic cholangiocarcinoma, iCC) at 1-month, 3–6-month and then at regular-6-month intervals. To identify predictive variables associated with not achieving or losing complete response two mixed-effects multivariable logistic regression models were constructed.

**Results:**

Sixty-three patients (40/23, male/female) with primary liver malignancies (HCC = 49; iCC = 4) and metastasis (n = 10) were treated. Median diameter of target lesion was 4.5 cm (range 3.0–7.0 cm). The median follow-up time was 9.2 months. At one-month follow-up, 79.4% of patients presented with a complete response and the remaining 20.6% were partial responders. At the 3–6-month follow-up, reached by 59 of the initial 63 patients, 83.3% showed a sustained complete response, while 10.2% had a partial response and 8.5% a local recurrence. At the last follow-up, 69.8% of the lesions showed a complete response. The initial diameter of the target lesion ≥ 5.0 cm was the only independent variable associated with the risk of failure in maintaining a complete response at 6 months (OR = 8.58, 95% CI 1.38–53.43; *P* = 0.02).

**Conclusion:**

Percutaneous thermal segmentectomy achieves promising oncological results in patients with tumors > 3.0 cm, with tumor dimension ≥ 5.0 cm being the only risk factor associated with the failure of a sustained complete response.

## Introduction

Percutaneous thermal ablation represents the unique curative locoregional procedure in the armamentarium of Interventional radiology. Its current applications and indications are limited by some technical constraints. According to the CIRSE standard of practice [1], a safety margin between 0.5 and 1 cm in all directions should be achieved to prevent the persistence of residual tissue or local recurrence, as surgical resection does. A single antenna does not permit to obtain appropriate safety margins in the treatment of lesions with a diameter > 3.0 cm, even when the newest ablation generators—that allow for larger necrotic areas—are employed.

Several technical approaches have been proposed to overcome this limitation in size. Firstly, single antenna multiple positioning has been suggested, but it has never turned into practice due to technical limitations such as charring of tissue, gas visualization impairment of proper antenna repositioning and technical limitations in covering the entire volume of the tumor plus ablation margins in three dimensions with sequential antenna positioning [2]. Recently, concomitant positioning of multiple antennas has been proposed as a valid alternative for ameliorating treatment response, raising the bar of clinical oncological performance in lesions greater than 3.0 cm [3–5]. Despite promising results, this technique remains limited to high-volume centers, with some adjunctive constraints related to the learning curve, costs, need for CT/Cone Beam CT (CBCT) or fusion-imaging guidance, navigation and robotic systems, and applicability to all lesions locations (i.e., subcapsular or exophytic).

An alternative historically proposed locoregional approach is the combination of TACE and ablation, (employing different TACE and ablation techniques in various orders and timings) to overcome the limited performance of stand-alone treatments for tumors larger than 3 cm. Since the late 90’s, attention has been focused on the execution of ablation under temporary occlusion of feeding vessels to boost the clinical performance of ablative procedures, such as radiofrequency and microwave ablation (MWA). This was applied in pig models through the Pringle maneuver [6–8], during balloon occlusion of the hepatic artery [9] or liver blood outflow occlusion [10], demonstrating larger ablated zones. Therefore, the first clinical experiences were in the early 2000s, when three distinct research groups demonstrated improved clinical performance when RFA was performed under occlusion [11–14] In these studies, occlusion was performed proximal (proper hepatic or right/left hepatic artery) or using a more complex technique due to the lack of a balloon microcatheter.

Finally, owing to the advent of MWA and the development of balloon microcatheters, which permits catheterization of smaller vessels, the concept of percutaneous thermal segmentectomy was proposed [15, 16]. This technique, consisting of a single-step combination of balloon-occluded MWA (bMWA) followed by balloon-occluded TACE (bTACE), permits the achievement of a wider and segmental necrotic area with a single antenna and a single energy delivery under temporary occlusion of the segmental artery.

The aim of this study was to report the mid-term clinical performance of a multicenter study of percutaneous segmentectomy in treating primary and secondary hepatic tumors greater than 3.0 cm.

## Material and methods

### Study design

This was a retrospective multicenter study, based on prospectively maintained databases from five Italian centers, of patients with primary or secondary hepatic tumors > 3.0 cm treated with percutaneous thermal segmentectomy. Indication for percutaneous thermal segmentectomy was given by the respective multidisciplinary tumor board for patients (age > 18 years) with tumors > 3 cm considered unfit for surgery; absolute contraindications for percutaneous thermal segmentectomy are the same as the ones for thermal ablation and TACE. Subcapsular location of the lesion was not considered a relative contraindication to percutaneous thermal segmentectomy, contrary to thermal ablation alone, since for this technique a perfect ballistic of the antenna is not needed due to the wider dimension of the necrotic area obtained through arterial occlusion [15].

The study followed the Strengthening the Reporting of Observational Studies in Epidemiology (STROBE) reporting guidelines [17]. All authors had access to the study data and reviewed and approved the final manuscript. The institutional review board of “University La Sapienza” (coordinating center) approved the study, which was conducted in accordance with both the Declarations of Helsinki and Istanbul, and written consent was obtained from all enrolled patients.

All consecutive adult patients, who underwent a percutaneous thermal segmentectomy for the treatment of a hepatic tumor larger than 3 cm in size were retrospectively enrolled in our study.

### Treatment’ technical aspects

All treatments were performed by an interventional radiologist in an angiographic suite, using a single-step approach. Antibiotic prophylaxis, patient monitoring and anesthesiologist assistance were performed routinely.

After catheterization of the hepatic artery via the radial or femoral route, liver vascular anatomy was assessed with Digital Subtraction Angiography (DSA) and CBCT acquired with a 4 or 5 Fr catheter. All centers acquired an arterial phase with the CBCT to identify all the lesion’s feeders and the best site to position and inflate the balloon microcatheter (Occlusafe, Terumo Europe NV, Leuven, Belgium): power injector parameters were set to 4 ml/s and intra-arterial injection lasted 15 s to maintain arterial tree enhancement during CBCT acquisition, that was set 8 s after the injection.

Selective catheterization was achieved with the balloon-microcatheter positioned in the vessel proximal to all the feeders of the tumor.

Then, the invasive arterial pressure at the tip of the microcatheter was measured.

US-guided MW antenna positioning was performed under local anesthesia or sedation according to requirements. Thereafter, the balloon was inflated to occlude the flow and obtain an arterial stump pressure drop (BOASP) and ablation was performed immediately after sedation.

Different ablation systems were employed across different centers (Tato, Terumo, Tokyo, Japan; Emprint, Medtronic, Minneapolis, USA; Amica, HS, Rome, Italy). Different power and timing were employed based on the vendor’s ablation chart. Considering that all the lesions treated with percutaneous thermal segmentectomy were beyond the threshold of 3 cm, in all cases the maximum time and power were set. At the end of the ablation, the antenna was withdrawn by performing track ablation.

With the balloon-microcatheter still inflated, bTACE was performed using 100-micron calibrated drug-eluting-microspheres (LifepearlTM, Terumo Europe NV, Leuven, Belgium) pre-loaded with 50 mg epirubicin/doxorubicin for HCC, iCC and breast metastases and 100 mg of irinotecan for mCRC and sarcoma metastases.

### Outcomes

The primary endpoint was to evaluate the mid-term oncological performance and to investigate the risk factors associated with not achieving a sustained radiologically evaluable complete response in the treated target lesion during follow-up.

### Variables, data collection and definitions

Preoperative data collected in the study included patient age in years, sex, type of liver tumor, site of the original tumor in case of metastatic disease, diameter of the target lesion, Child–Pugh score and model for end-stage liver disease (MELD) score in cases of underlying cirrhosis, multifocal or multilobar disease of the liver, number of lesions, distance from the target lesion to the major vessel (cm), distance from the target lesion to the liver capsule (cm), caliber of the major vessel (cm), and signs of hypervascularization of the target lesion at pre-treatment contrast-enhanced CT or MRI.

The modified Response Criteria in Solid Tumors (mRECIST) [18] were used to evaluate the oncological response of patients with HCC, while RECIST v1.1 [19] was used for other malignancies. During follow-up, the oncological response of the target lesion was evaluated on abdominal multiphasic CT or MRI performed at 1 month, at 3–6 month and then at regular 6-month interval. Oncological responses were also reported at the last available follow-up. Objective Response Rate (ORR, the cumulative sum of lesions in Complete Response—disappearance of the target lesion—and Partial Response—reduction of the dimension of the target lesion > 30%) and its duration (duration of response, DoR) were evaluated.

Considering the different nature and dimensions of the treated lesions, the oncological response was displayed for the entire population, for HCC only, for tumors between 3.0 and 5.0 cm and > 5.0 cm.

Adjunctive treatments for patients in whom a complete response was lost or not achieved were performed and recorded. Deaths and orthotopic liver transplantations (OLT) were recorded during the follow-up.

### Statistical analysis

Continuous variables are reported as medians and the first and third quartile (Q1-Q3). Categorical variables are described as numbers and percentages. Missing data were not observed for the covariates used to construct the models. Therefore, no data interpolation was required.

Due to the small sample size and the risk of collinearity phenomena, the variables to be used for constructing the models were preliminarily selected using a Least Absolute Shrinkage and Selection Operator (LASSO) regression (stepwise regression with backward Wald elimination), with the intent of creating a parsimonious model in terms of the number of covariates [20]. Given the risk of data separation within the five centers involved, a multivariable logistic regression model with mixed effects was created, in which the center was incorporated into the model as a cluster-specific random effect variable [21].

Ten different variables were initially tested, with those with a *P* value < 0.20 selected for constructing the models: patient age (years), male sex, HCC, diameter of the target lesion ≥ 5.0 cm, monofocal lesion, monolobar lesion, number of lesions, distance from the major vessel (cm), distance from the liver capsule (cm), caliber of the major vessel (cm).

Two mixed-effects multivariable logistic regression models were constructed to identify the predictive variables for the risk of complete response failure at one and six months after percutaneous thermal segmentectomy. Odds ratios (OR) and 95% confidence intervals (95%CI) are reported as significant variables.

Survival analyses were performed using the Kaplan–Meier method, and the log-rank test was used to compare the survival rates. Statistical significance was set at *P* < 0.05. Statistical analyses were performed using the SPSS statistical package (version 27.0, SPSS Inc., Chicago, IL, USA).

## Results

### Baseline characteristic

The final population included 63 patients treated between September 2019 and March 2023 at five Italian centers.

The demographics characteristics of the study population are illustrated in Table 1. The median age of the population was 68 years, with a higher prevalence of males (63.5 vs. 36.5%). In the majority of cases, HCC was treated (49/63, 77.8%), followed by metastatic disease (10/63, 15.9%) and iCCA (4/63, 6.3%). In 33.3% of the patients, previous hepatic locoregional therapies had already been performed, while all target lesions were naive to locoregional therapies.

**Table 1:** Demographics characteristics

Variables	Entire population (N = 63)
	Median (Q1–Q3) or n (%)
Age, years	68 (62–76)
Male/female	40/23 (63.5/36.5)
*Tumoral disease* HCC iCCA metastases*	49 (77.8) 4 (6.3) 10 (15.9)
*Diameter target lesion, cm* > 5 cm	4.5 (3.7–5.2) 26/63 (41.3)
Number of lesions	1 (1–2)
Single/multiple tumors	47 (74.6)/16 (25.4)
Monolobar/Bilobar disease	54 (85.7)/9 (14.3)
Distance from the major vessel, cm	0.3 (2–6)
Distance from the liver capsule, cm	4 (0–1.0)
Caliber of the adjacent major vessel, cm	0.6 (0.4–0.7)
Hypervascularity of the target lesion	43 (68.3)
Naïve patient	42 (66.7)
*In cirrhotic patients (n* = *49)* Child–Pugh Score A/B/C MELD score	42/7/0 (85.7/14.3) 10 (8–10)

*Primary tumors for metastasis: colorectal (n = 4), lung neuroendocrine (n = 2), sarcoma (n = 1), breast (n = 1), esophagus (n = 1), tongue (n = 1)

Q1–Q3, first-third quartile; HCC, hepatocellular carcinoma; iCCA, intrahepatic cholangiocarcinoma; MELD, model for end-stage liver disease

The median diameter of the target lesion was 4.5 cm, with 41.3% (26/63) of cases presenting a lesion ≥ 5.0 cm and 58.7% (37/63) with a lesion within 3.0 and 5.0 cm. In 74.6% of cases the tumor was monofocal and target lesion was hypervascular in 68.3% of cases.

### Oncological performance

During the entire follow-up, six patients underwent OLT and six died of tumor-related or -unrelated causes. The median follow-up period for the entire cohort was 9.2 months (Q1–Q3 = 6.0–12.6).

Oncological response rates evaluated at each timeline and stratified according to dimensions and histology (3.0–5.0 cm, > 5.0 cm, HCC) are reported in Table 2.

**Table 2:** Oncological response according to mRECIST and RECIST v1.1 at 1 month, 3–6 months and afterward every 6 months

	1-month follow-up			3–6-months follow-up				6–12-months follow-up				12–18-months follow-up			
	CR	PR	OR	CR	PR	LR	OR	CR	PR	LR	OR	CR	PR	LR	OR
3–5 cm (n = 37)	89.2% (33/37)	10.8% (4/37)	**100%**	90.9% (30/32)	3.0% (1/32)	6.1% (2/32)	93.9%	83.3% (20/24)	4.2% (1/24)	12.5% (3/24)	87.5%	78.6% (11/14)	0	21.4% (3/14)	78.6%
> 5 cm (n = 26)	65.4% (17/26)	34.6% (9/26)		69.3% (18/26)	19.2% (5/26)	11.5% (3/26)	88.5%	80.0% (16/20)	5.0% (1/20)	15.0% (3/20)	85.0%	50% (6/12)	0	50% (6/12)	50%
HCC (n = 49)	*75.5% (37/49)*	*24.5% (12/49)*		*80.4% (37/43)*	*11.1% (6/43)*	*5.6% (3/43)*	*91.5%*	*82.9% (29/35)*	*2.4% (2/35)*	*14.6% (4/35)*	*85.3%*	*61.9% (13/21)*	*0*	*38.1% (8/21)*	*61.9%*
Entire cohort (n = 63)	**79.4%** (50/63)	**20.6%** (13/63)		**83.3%** (48/59)	**10.2%** (6/59)	**8.5%** (5/59)	**93.5%**	**81.8%** (36/44)	**2.3%** (2/44)	**13.6%** (6/44)	**84.1%**	**65.4%** (17/26)	**0**	**34.6%** (9/26)	**65.4%**

*CR* complete response, *PR* partial response, *LR* local recurrence, *OR* objective response

All patients reached the one-month follow-up; at this timeframe, 79.4% (50/63) of the lesions presented a complete response, with only 20.6% (13/63) of the patients showing a partial response. In these latter cases, the median tumor diameter was 5.8 cm, and the residual tumor was 2.2 cm.

The 3–6-month follow-up was not reached by 4/63 patients: 1/63 died of tumor-unrelated causes, 2/63 underwent OLT and 1/63 was lost at follow-up. A complete response was observed in 81.4% of the patients (48/59), while 11.0% (6/59) and 9.3% (5/59) of the patients experienced a partial response or local recurrence, respectively.

At the last available follow-up, 69.8% of the lesions (44/63) showed a complete response, 7.9% (5/63) a partial response and 22.2% (14/63) a local recurrence.

The median local recurrence diameter was 2.5 cm (Q1–Q3 = 1.6–3.2). During the follow-up period, 11 retreatments for local recurrence or partial response with locoregional therapies were performed, achieving a complete response in 8/11 patients and a partial response in 3/11.

In the subgroup of tumors with dimensions between 3.0 and 5.0 cm, complete response rates at 1, 3–6, 6–12 and 12–18 months were 89.2%, 90.9%, 83.3% and 78.6, respectively, with local recurrence occurring at 3–6, 6–12 and 12–18 months in 6.1%, 12.5% and 21.4% of patients.

ORR was 100% at one-month, with a median DoR of 9.1 months for the entire population and 9.3 months for the HCC subpopulation. In the 52/63 patients who underwent imaging at least 6 months after the treatment (4 patients died, 4 underwent OLT and 3 were lost at follow-up), a DoR > 6 months was achieved in 98.1% of the cases. One patient with HCC experienced a recurrence at 3.8 months (thus leading to a DoR > 6 months in the population of HCC at a rate of 97.6%) (Table 3).

**Table 3:** Objective response rates and its duration

*Objective response rates (percentage)*	
1 month	100%
3–6 months	93.50%
6–12 months	84.10%
12–18 months	65.40%
*Duration of response (DoR) (months, median Q1–Q3)*	
Entire population	9.1 (6–12.1)
HCC	9.3 (6–12.3)
DoR > 6 months * *(n, percentage)*	98.1% (51/52)
*Response at last available follow-up*	
All population	
Complete response	69.8% (44/63)
Partial response	7.9% (5/63)
Local recurrence	22.2% (14/63)
3.0–5.0 cm	
Complete response	78.4% (29/37)
Partial response	5.4% (2/37)
Local recurrence	16.2% (6/37)
> 5.0 cm	
Complete response	57.7% (15/26)
Partial response	11.5% (3/26)
Local recurrence	30.8% (8/26)

*52/63 patients underwent imaging at least 6 months after the treatment (four patients died, four underwent OLT, three were lost at follow-up)

### Factors associated with not achieving or maintaining a complete response

**Fig. 1:**
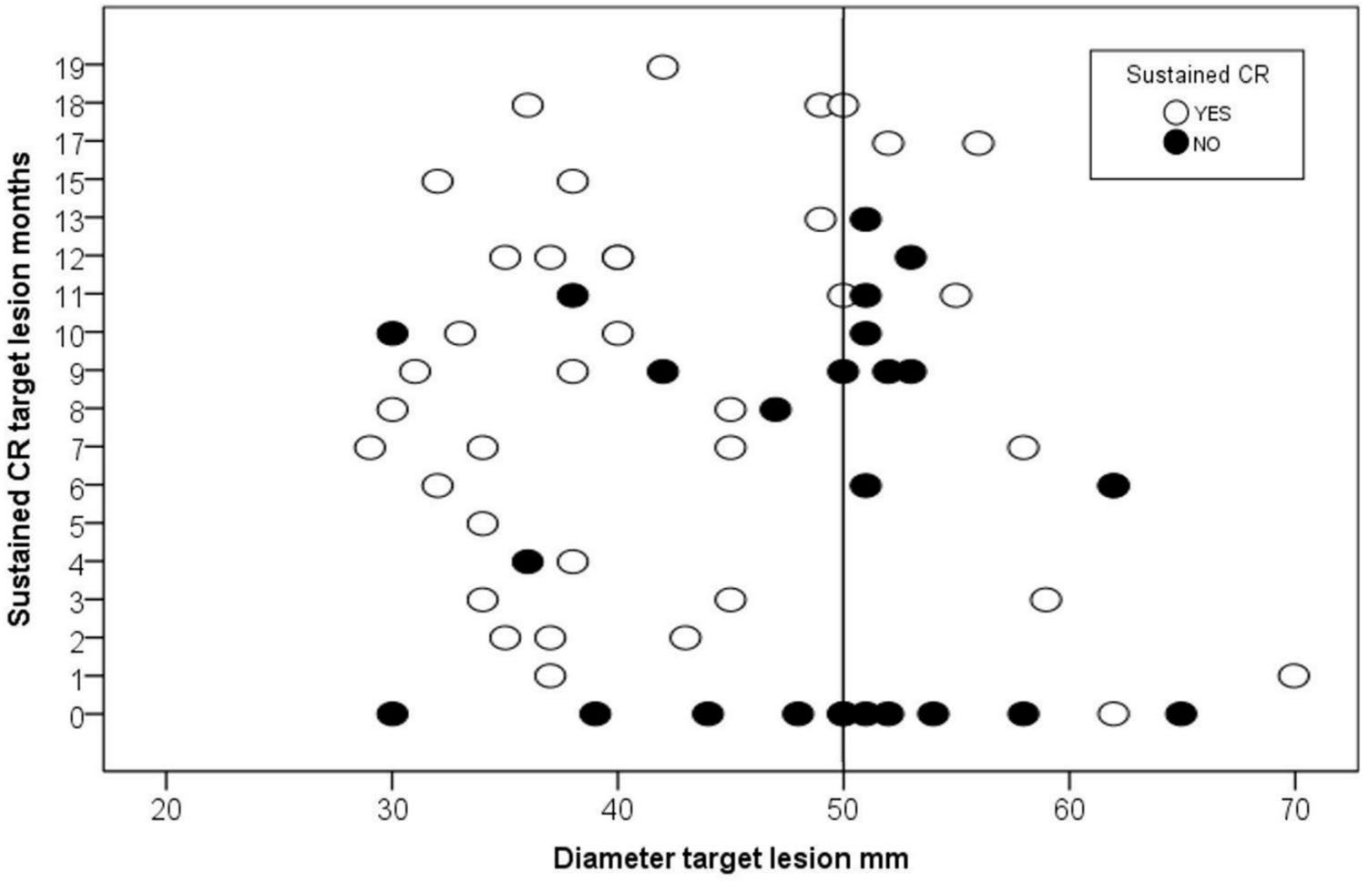
Distribution of the population according to the initial diameter of the target lesion and the time of complete response in months. Black circles: patients with loss of complete response; white circles: patients with sustained complete response at last follow-up

In Fig. 1, the distribution of cases with sustained complete response is reported, with the cases stratified according to the initial dimension of the target lesion and duration of complete response. Two clusters of complete response failure were observed, one corresponding to the initial failure of percutaneous thermal segmentectomy (partial response one month after treatment), and one approximately nine months after the treatment.

The potential risk factors for failure of the complete response in the target lesion were investigated at two different time-points, namely, one month and six months after the treatment. No relevant risk factors were identified when a time horizon of one month was adopted, as shown in Table 4.

**Table 4:** Risk factors for the failure of sustained complete response after treatment at one month and at 6 months

Variables	Beta	SE	Wald	OR	95% CI		*P*
					Inferior	Superior	
*Failure of complete response at 1 month*							
Hepatocellular cancer	0.94	1.13	0.69	2.56	0.28	23.37	0.41
Caliber of the major adjacent vessel in cm	− 0.19	0.19	1.01	0.82	0.57	1.20	0.32
Diameter target lesion ≥ 5.0 cm	1.00	0.71	2.01	2.73	0.68	10.91	0.16
Constant	− 1.70	1.61	1.12	0.18	−	−	0.29
*Hosmer–Lemeshow test: 0.7*							
Failure of complete response at 6 months							
Diameter target lesion ≥ 5.0 cm	2.15	0.93	5.30	8.58	1.38	53.43	0.02
Hypervascular lesion	− 1.26	0.93	1.84	0.28	0.05	1.76	0.18
Constant	0.93	0.46	4.09	2.52	−	−	0.04
Hosmer–Lemeshow test: 0.90							

Variables initially included in the model and then excluded using a backward wald method: patient age in years, male sex, hepatocellular cancer, monofocal lesion, monolobar lesion, number of lesions, distance from the major vessel in cm, caliber of the major vessel in cm, distance from the Glissonian capsule in cm

*SE* standard error, *OR* odds ratio, *CI* confidence intervals

When the model for the risk at six months was built, the initial diameter of the target lesion ≥ 5.0 cm was the unique independent variable for the risk of complete response failure (OR = 7.06 [95% CI 1.06–47.10; *P* = 0.04]) (Table 4).

**Fig. 2:**
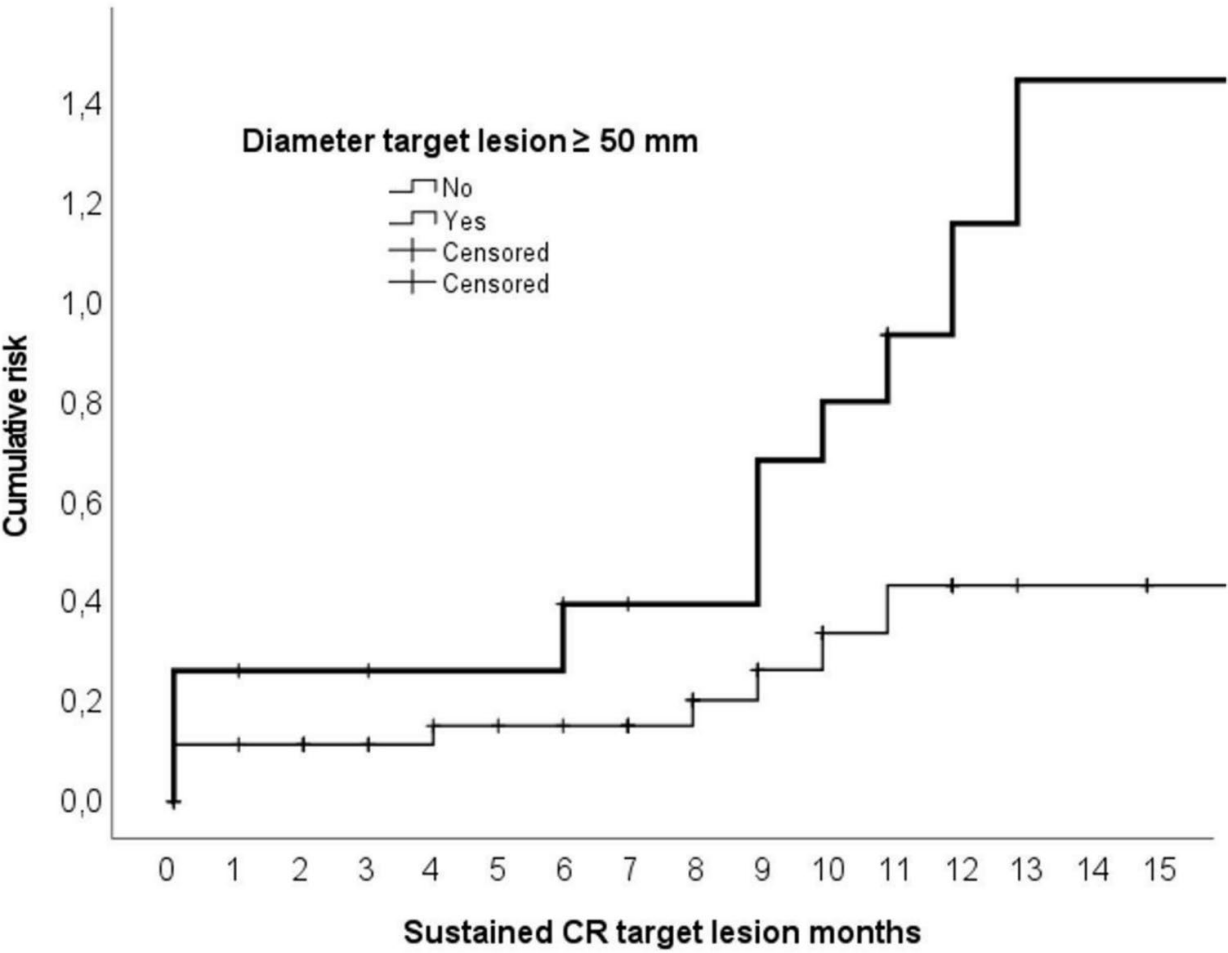
Estimation of the cumulative risk of local recurrence in patients according to the initial diameter of the target lesion ≥ 5 cm

At Kaplan–Meier analysis, the initial diameter of the target lesion ≥ 5.0 cm effectively stratified the investigated population for the cumulative risk of complete response failure (log-rank = 0.02), with a spread between the two curves observed mainly starting nine months after the treatment (Fig. 2).

## Discussion

**Fig. 3.**
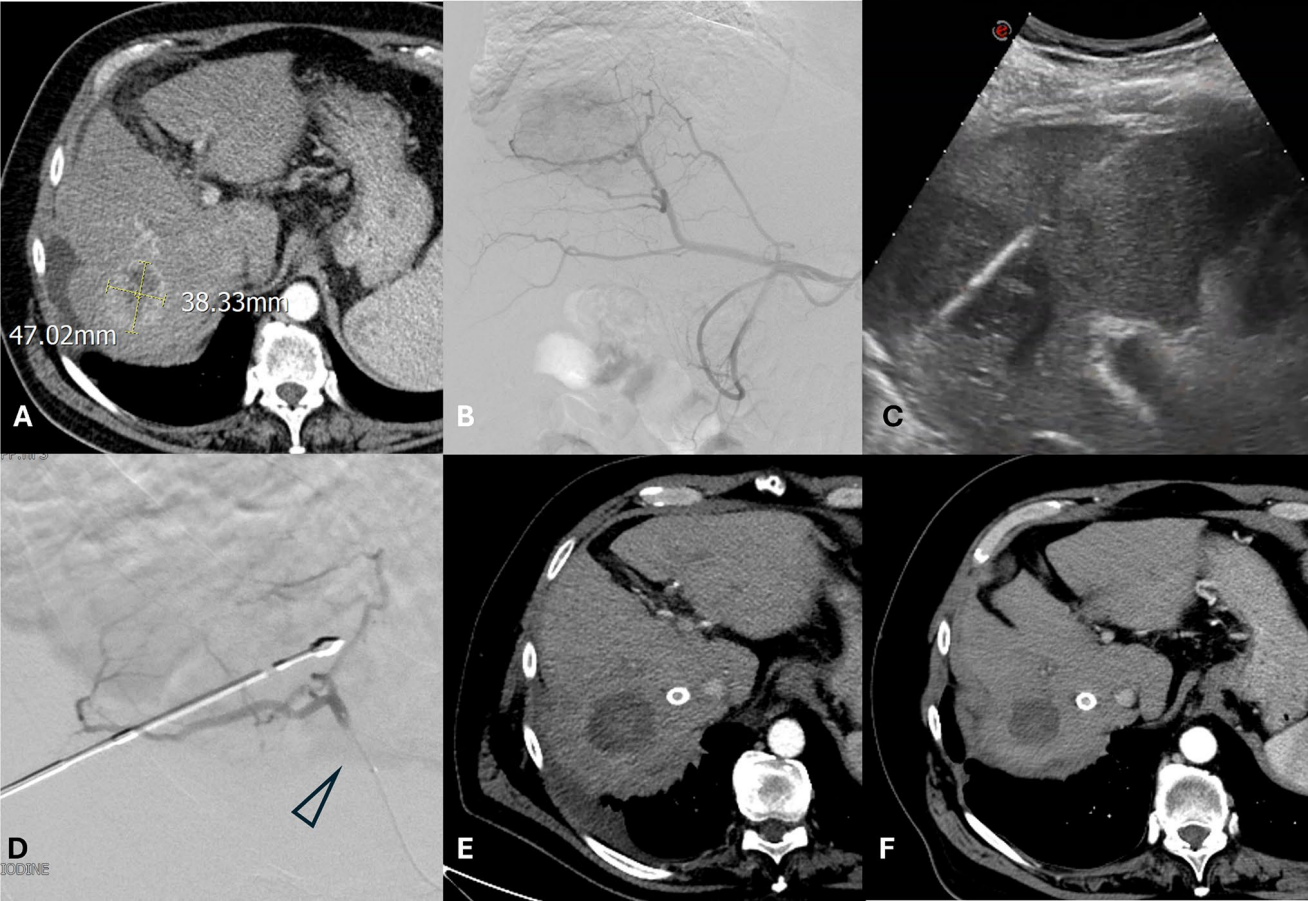
62 years old man with alcoholic cirrhosis in OLT waiting list, with portal and mesenteric vein thrombosis, for a 47-mm HCC located in segment VIII, partially necrotic and hypervascular at the CT arterial phase (**a**). Percutaneous thermal segmentectomy was performed as bridging two weeks before TIPS creation. Digital subtraction arteriography (**b**) confirmed the hypervascular lesion; MW antenna was positioned under US guidance (**c**) and ablation performed after micro-ballon catheter inflation (arrowhead, **d**). One month CT follow-up, performed after TIPS placement, showed Complete response of the lesion, sustained up to 15 months (**f**)

**Fig. 4.**
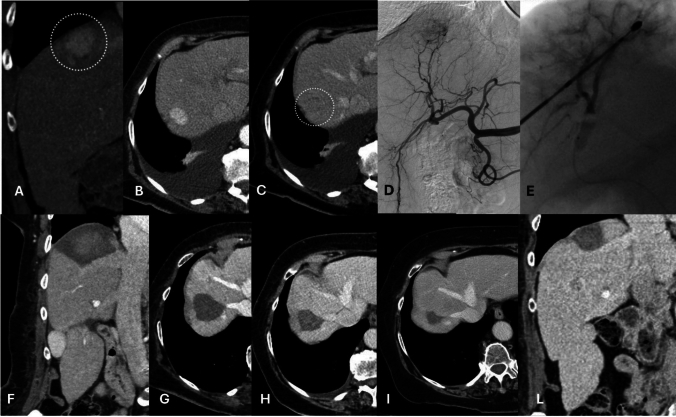
76 years old cirrhotic female, with a not capsulated 32 mm HCC in segment VII, hypervascular in the arterial phase (MIP 3 mm coronal reconstruction: **a**, and axial: **b**), with late wash-out **c** showed a (dotted circle), confirmed at the angiography (trans-femoral, anteroposterior projection digital subtracted angiography, **d** and treated **e** with percutaneous thermal segmentectomy. Post-procedural 24 h CT (venous phase coronal plane, **f**) showed a triangular necrotic area shape that encompassed all the HCC, thus configuring a complete response. The complete response was maintained at the subsequent follow-up, performed at one (**g**), twelve (**h**), twenty-four (**i**) and forty (**l**) months

This multicenter analysis of oncological results of percutaneous thermal segmentectomy for the treatment of hepatic tumors > 3.0 cm, demonstrates its capability to achieve an objective response rate of 100% at one month, with 79.4% of these obtaining a complete response, which was sustained until 12–18 months in 78.6% of nodules between 3 and 5 cm (as shown in Figs. 3 and 4).

The oncological performance of percutaneous thermal segmentectomy for different types of malignancies is not easily comparable to the standard of care; therefore, a sub-analysis of its performance for HCC has been conducted. Tumor diameter > 3 cm is a well-known negative prognostic factor for achieving an objective response and reducing local recurrence -even after curative treatment such as surgery or thermal ablation [22]. According to the BCLC guidelines [23], options for unresectable HCC > 3.0 cm are TACE or transarterial radioembolization (TARE) when no other solutions are feasible.

Regarding TACE, one of the most recent studies is a pooled multicentric analysis of 557 patients conducted by Veloso Gomes et al. [24], which demonstrated best complete tumor response rates of 60.14% and partial response rate of 27.11%, which were inferior to those observed after percutaneous thermal segmentectomy. Additionally, this TACE population presented a mean tumor diameter < 3.0 cm, compared to our median diameter of 4.5 cm. In clinical practice, balloon-occluded TACE has been proposed as an effective tool for achieving better oncological responses. Golfieri et al. [25] recently reported in a multicenter European study similar short-term ORR (90.1% vs. 100% for percutaneous thermal segmentectomy) but with lower complete responses (59.3% vs. 79.4%), although data on long-term oncological response are not reported in this study. Gwong et al. [26] obtained better local recurrence rates with conventional balloon occluded TACE compared to those observed with percutaneous thermal segmentectomy (1.5% at 6 months, 14.2% at 12 months, 21% at 24 and 36 months), but treated smaller lesions (mean diameter 3.1 cm, range of 1–5 cm).

Transarterial radioembolization is the second option and tool in the armamentarium of locoregional therapies for HCC in early or intermediate stages, newly introduced in BCLC guidelines [23]. The LEGACY trial [27] is one of the principal studies that allowed the introduction of this technique in clinical guidelines. This multicenter retrospective study included 162 patients treated with Yttrium-90 radioembolization, evaluating oncological response through ORR, DoR and localized mRECIST. The best ORR was 88.3% (84% CR and 4.3% PR), with 76.1% exhibiting DoR ≥ 6 months. Those results are inferior to those obtained in this study with a single step procedure, where the best ORR was 100% and DoR ≥ 6 months was appreciable in 97.6% in the HCC. In the LEGACY trial, no progression was described, which might be correlated with the smaller median dimensions of the tumors treated. It is worth noting that the results in the LEGACY trial were achieved in a population with median tumor size of 2.7 cm -most being < 3.0 cm (61.7%)—and requiring two treatment sessions in 19.8% of the patients. Kim et al. [28] prospectively investigated radiation segmentectomy with curative intent for the treatment of HCC < 3 cm; this group treated 29 patients, obtaining an initial complete response in 83% of the lesions and partial response in 17% of patients, results inferior to those obtained in our series in tumors between 3 and 5 cm, where the initial complete response rate was 89.2% and the partial response of 10.8%. Additionally, it is worth noting that oncological performance of percutaneous thermal segmentectomy can be evaluated at the first one-month follow-up, allowing for an early assessment, which is crucial in the context of OLT (occurred in six patients in this study), rather than waiting 3 to 6 months after TARE, a time considered necessary for the disappearance of hyperemia caused by inflammation, which makes follow-up scans less reliable [29, 30].

Solutions not covered by BCLC guidelines but worldwide utilized by interventional radiologists in clinical practice include combined treatment (TACE + ablation) and multiple antenna positioning.

For combined treatment, among the ablation techniques proposed in combination with TACE, MWA achieved the best clinical performance [31]. Zhao et al. [32] in a recent meta-analysis on MWA + TACE, reported OR rates of 87.7%, lower than the 100% rates obtained in our series with single step percutaneous thermal segmentectomy, despite the studies included in the meta-analysis also encompassing tumors < 3 cm. Moreover, Yu et al. [33] performed a propensity matched study on the safety and effectiveness of combined therapy (standard MWA + TACE) vs. TARE for treating naïve, unresectable, solitary HCC ≥ 3 cm, and found no statistically significant difference in oncological performance.

Recently percutaneous thermal ablation for colorectal metastasis has been shown, in a phase III randomized trial, to be an alternative to surgery in terms of overall survival and local tumor recurrence for lesions below 3 cm, but with fewer adverse events and lower costs [34]. Beyond the limit of 3 cm, as for HCC, technical constraints remain. The results of the present series show promising tumor control even when targeting mCRC and other types of secondary liver tumors, especially when between 3 and 5 cm, thus proposing an easily repeatable locoregional strategy that could be helpful in the setting of inoperable oligometastatic disease or oligo progression.

Multi antenna ablation has been proposed in few centers worldwide for liver tumors—primary or secondary—that exceed the 3 cm threshold [3–5]. Andresciani et al. [3] recently reported their experience with double antenna simultaneous ablation in a similar population (4 ± 1 cm, range 2.1–7.0 cm) but with 18% of the lesions < 3.0 cm, obtaining 88.9% of complete response at 12 months versus 81.8% in our series. Additionally, double antenna-positioning requires operator's experience for precise placement with parallelism and distance between the antennas, under ultrasound or CT/CBCT guidance, limiting its potential application in subcapsular lesions.

Analysis of correlation between negative prognostic factors for not achieving a complete response at first follow-up (caliber of the major vessel, diameter of the target lesion and subcapsular location), failed to demonstrate a correlation, while the initial diameter of the target lesion ≥ 5.0 cm emerged as the sole independent variable for loss of the CR, that occurred at a median time of 9 months. These findings indicate the best oncological performance of percutaneous thermal segmentectomy in tumors between 3.0 and 5.0 cm, despite its employment in tumors > 5.0 cm allowed to obtain an objective response rate of 100% and small-dimensional recurrence or partial responses, which are easily treatable. In fact, the median diameter of local recurrence/partial response was 2.5 cm, allowing for retreatment with locoregional therapies leading to a final CR in 72.8% of the lesions and a PR in the remaining 27.2%. Moreover, loss of CR after initial treatment was not correlated with the hypervascular/hypovascular nature of the target lesion. This seems to suggests that the clinical performance of percutaneous segmentectomy relies primarily on temporary occlusion of the segmental arterial pedicle, regardless of lesion’s characteristics, thus finding an application even for hypovascular lesion—very frequent in metastases—that are not easily treatable with exclusively transcatheter therapies. However, more data in future studies are needed to clearly demonstrate the potential advantages of the use of balloon microcatheter in hypovascular tumors, mainly related to the vascular flow diversion created and flow redistribution obtained.

Finally, percutaneous thermal segmentectomy is a single-step procedure performed with an armamentarium that is already available to interventional radiologists worldwide, requiring no specific training. Since this procedure is carried out entirely by one department without the need to involve other services, it could be a solution for hospitals without nuclear medicine departments or in developing countries. Although not the objective of this study, a significant potential reduction in overall procedural costs might be achieved compared to the current standard of care, apparently without compromising oncological outcomes.

This study has some limitation, the main one being the absence of a control group; moreover, the retrospective and multicentric study design of the study limited the ability to remove potential selection confounders and introduced potential confounders from the different strategies and managements approaches adopted in the various centers. Lastly, the study has a limited sample size and relatively short median follow-up time. All these limitations are related to the novelty of this technique compared to other standards of practice. A longer enrollment and follow-up are needed to further confirm our initial results, which lay the groundwork for a direct comparison with radioembolization and surgery for tumors between 3 and 5 cm.

## Conclusion

Percutaneous thermal segmentectomy is globally available alternative intervention that showed promising mid-term follow-up results for lesions > 3 cm, with its best performance between 3 and 5 cm, suggesting its potential role as an alternative to the oncological standard of care.

## Data Availability

All data generated or analyzed during this study are included in this article. Further enquiries can be directed to the corresponding Author.

## Abbreviations

MWA: Microwave ablation
BMWA: Balloon-occluded MWA
BTACE: Balloon-occluded TACE
HCC: Hepatocellular carcinoma
ICC: Intra-hepatic cholangiocarcinoma
CR: Complete response
PR: Partial response
ORR: Objective response rate
LR: Local recurrence
DoR: Duration of response
mRECIST: Modified response criteria in solid tumors
OLT: Orthotopic liver transplantations
